# The Biting Midge *Culicoides sonorensis* (Diptera: Ceratopogonidae) Is Capable of Developing Late Stage Infections of *Leishmania enriettii*

**DOI:** 10.1371/journal.pntd.0004060

**Published:** 2015-09-14

**Authors:** Veronika Seblova, Jovana Sadlova, Barbora Vojtkova, Jan Votypka, Simon Carpenter, Paul Andrew Bates, Petr Volf

**Affiliations:** 1 Department of Parasitology, Faculty of Science, Charles University, Prague, Czech Republic; 2 Vector-borne Viral Diseases Programme, The Pirbright Institute, Pirbright, Surrey, United Kingdom; 3 Division of Biomedical and Life Sciences, School of Health and Medicine, Lancaster University, Lancaster, United Kingdom; Liverpool School of Tropical Medicine, UNITED KINGDOM

## Abstract

**Background:**

Despite their importance in animal and human health, the epidemiology of species of the *Leishmania enriettii* complex remains poorly understood, including the identity of their biological vectors. Biting midges of the genus *Forcipomyia (Lasiohelea)* have been implicated in the transmission of a member of the *L*. *enriettii* complex in Australia, but the far larger and more widespread genus *Culicoides* has not been investigated for the potential to include vectors to date.

**Methodology/Principal Findings:**

Females from colonies of the midges *Culicoides nubeculosus* Meigen and *C*. *sonorensis* Wirth & Jones and the sand fly *Lutzomyia longipalpis* Lutz & Nevia (Diptera: Psychodidae) were experimentally infected with two different species of *Leishmania*, originating from Australia (*Leishmania* sp. AM-2004) and Brazil (*Leishmania enriettii*). In addition, the infectivity of *L*. *enriettii* infections generated in guinea pigs and golden hamsters for *Lu*. *longipalpis* and *C*. *sonorensis* was tested by xenodiagnosis. Development of *L*. *enriettii* in *Lu*. *longipalpis* was relatively poor compared to other *Leishmania* species in this permissive vector. *Culicoides nubeculosus* was not susceptible to infection by parasites from the *L*. *enriettii* complex. In contrast, *C*. *sonorensis* developed late stage infections with colonization of the thoracic midgut and the stomodeal valve. In hamsters, experimental infection with *L*. *enriettii* led only to mild symptoms, while in guinea pigs *L*. *enriettii* grew aggressively, producing large, ulcerated, tumour-like lesions. A high proportion of *C*. *sonorensis* (up to 80%) feeding on the ears and nose of these guinea pigs became infected.

**Conclusions/Significance:**

We demonstrate that *L*. *enriettii* can develop late stage infections in the biting midge *Culicoides sonorensis*. This midge was found to be susceptible to *L*. *enriettii* to a similar degree as *Lutzomyia longipalpis*, the vector of *Leishmania infantum* in South America. Our results support the hypothesis that some biting midges could be natural vectors of the *L*. *enriettii* complex because of their vector competence, although not *Culicoides sonorensis* itself, which is not sympatric, and midges should be assessed in the field while searching for vectors of related *Leishmania* species including *L*. *martiniquensis* and "*L*. *siamensis*".

## Author Summary

This study investigates the laboratory infection of two species of *Culicoides* biting midges (Diptera: Ceratopogonidae) and one species of sand fly (Diptera: Psychodidae) with two species of *Leishmania*. These members of the *L*. *enriettii* complex were demonstrated to colonize the stomodeal valve of *Culicoides sonorensis* following membrane feeding on blood-parasite mixtures or direct feeding on guinea pigs that demonstrated clinical signs of infection. In contrast, three other species of *Leishmania* that are known to be transmitted by sand flies failed to successfully develop in *C*. *sonorensis*. A sand fly species which is highly permissive to *Leishmania* infection, *Lu*. *longipalpis*, a widespread vector of *L*. *infantum* in Latin America, was found to support only moderate infections of *L*. *enriettii* from Brazil and *Leishmania* sp. AM-2004 from Australia. In addition to establishing a suitable laboratory model for infection of *Culicoides* with *L*. *enriettii*, successful infection of *C*. *sonorensis* highlights that vectors other than sand flies should be considered as part of epidemiological studies on parasites belonging to the *L*. *enriettii* complex.

## Introduction

The leishmaniases are widespread protozoan diseases with dermal or visceral clinical symptoms that affect humans and animals worldwide. Members of the genus *Leishmania* (Trypanosomatidae: Kinetoplastida) follow a digenetic life cycle, alternating between a vertebrate host and insect vector. To date, phlebotomine sand flies are considered the only proven vectors responsible for maintenance of the life cycle and transmission of these parasites. The *Leishmania* species infecting humans comprise about 20 species, mostly belonging to the subgenera *L*. (*Leishmania*) and *L*. (*Viannia*) [1].

Reservoir hosts may be human in some cases (anthroponotic transmission), but for the majority of *Leishmania* species infecting humans the reservoirs are domestic or wild animals (zoonotic transmission). Most experts studying sand fly-*Leishmania* interactions accept six classical criteria for vector incrimination [1,2] that would ideally be satisfied to fully prove vector status: 1, there is a strong ecological association between the vector and the reservoir host; 2, parasites are isolated and/or typed from wild caught vectors not containing recent blood meals and are shown to be identical to those in the reservoir host; 3, infections in such wild caught vectors exhibit parasites in the anterior midgut, on the stomodeal valve and the presence of metacyclic promastigotes, or such development beyond the blood meal can be demonstrated by experimental infection of the vector using laboratory colonies; 4, the vector is attracted to and bites the reservoir host; 5, the vector can be infected by biting and feeding on the reservoir host or an equivalent laboratory model (xenodiagnosis); 6, experimental transmission by bite is achieved to the reservoir or an equivalent laboratory model. However, whilst desirable, rarely are all these criteria satisfied before conclusions are drawn about the identity of *Leishmania* vectors. Outbreaks of known species in new foci and newly discovered species are particularly problematic as in such cases the reservoir may be uncertain or completely unknown, making the testing of many of these criteria difficult.

The epidemiology of leishmaniases caused by a group of species called the *L*. *enriettii* complex is poorly understood, but is becoming increasingly important to human health. According to phylogenetic studies the *L*. *enriettii* complex occupies a position basal to all other euleishmania species, but falls outside the established subgenera *Leishmania* and *Viannia* [3–7]. The first described species within the complex, *L*. *enriettii*, was isolated from the skin of domestic guinea pigs (*Cavia porcellus*) in Paraná State, Brazil [8–10] and a second species (currently unnamed, here termed *Leishmania* sp. AM-2004) was more recently isolated from red kangaroos in Australia [11]. These sporadic infections of guinea pigs and kangaroos were characterized by occurrence of tumour-like skin lesions on the ears, nose, feet and testicles in animals [8–10], but both species appear to be non-pathogenic to humans. However, the *L*. *enriettii* complex was also recently extended to include three species known to cause clinical disease in humans: *L*. *martiniquensis* from Martinique (Caribbean island) and Thailand [7,12]; a second species from Thailand recorded as "*L*. *siamensis*" [13]; and another new species from Ghana [14]. ("*L*. *siamensis*" has not been formally described so is used in quotation marks). In addition, DNA samples from cutaneous lesions in horses and cattle in Central Europe [15,16] and the USA [17] appear to be identical to *L*. *martiniquensis* [7]. Human infections with *L*. *martiniquensis* manifest clinically as cutaneous [12,18] or visceral disease [7,19], "*L*. *siamensis*" presented as mixed cutaneous and visceral disease [13], and in Ghana the disease has only been found in the cutaneous form [14].

Suspected vectors of the *L*. *enriettii* complex include a variety of sand fly and non-sand fly Diptera. In Brazil, *Lutzomyia monticola* was suggested as a possible vector for *L*. *enriettii* [9], although no definitive studies have been conducted [20]. Candidate vectors of *L*. *martiniquensis* include *Lutzomyia atroclavatus* and *Lu*. *cayennensis*, since these are the only known sand fly species on Martinique island [12]. In Thailand, *Leishmania* DNA was found in *Sergentomyia* species, namely *Sergentomyia gemmea* [21,22] and *S*. *barraudi* [22], although *Sergentomyia* species are not usually regarded as vectors for human-infective *Leishmania*. In contrast, in Australia, day-feeding biting midges of the genus *Forcipomyia* (*Lasiohelea*) were implicated as vectors of cutaneous leishmaniasis caused by *Leishmania* sp. AM-2004 in red kangaroos and other macropods [5,11,23]. Microscopical examination revealed that *Forcipomyia* produced late stage *Leishmania* infection of high intensities including colonization of the stomodeal valve, the presence of material resembling promastigote secretory gel (PSG) [24] and promastigotes with morphology of infectious metacyclic stages [5]. This evidence for midge-transmission of *Leishmania* sp. AM-2004 is compelling, and the strongest vector incrimination for any member of the *L*. *enriettii* complex, but is not conclusive as a number of the criteria set out above are yet to be satisfied or tested.

The aim of this study was to evaluate the possibility that *L*. *enriettii* is also midge-transmitted, as indicated for the related species *Leishmania* sp AM-2004. Direct testing of this hypothesis using wild caught midges from Brazil is currently not feasible, as there is no information on likely midge vectors or colonised insects from Brazil. Therefore, the vector competences of two species of midge available in established colonies were assessed, *Culicoides* (*Monoculicoides*) *sonorensis* and *C*. (*M*.) *nubeculosus*. Neither of these can be the true vector of *L*. *enriettii* as they are not sympatric, *C*. *sonorensis* is a north American species [25] and *C*. *nubeculosus* is European [26], but both are model systems that have been used to study a wide variety of arbovirus strains and species [25–27]. Infections of *L*. *enriettii* in these two midge species were generated by membrane feeding and compared with those produced in the neotropic sand fly *Lutzomyia longipalpis*, which is highly permissive for all *Leishmania* species tested to date [28]. To provide a parasite control, parallel infections in all three insects were also performed with *Leishmania* sp. AM-2004. These experiments were complemented by the use of guinea pigs (*Cavia porcellus*) and golden hamsters (*Mesocricetus auratus*) experimentally infected with *L*. *enriettii* in xenodiagnosis experiments with *C*. *sonorensis* and *Lu*. *longipalpis*, testing the ability of these insects to acquire infections by feeding on these mammalian hosts.

## Materials and Methods

### Ethical statement

Animals were maintained and handled in the animal facility of Charles University in Prague in accordance with institutional guidelines and Czech legislation (Act No. 246/1992 and 359/2012 coll. on Protection of Animals against Cruelty in present statutes at large), which complies with all relevant European Union and international guidelines for experimental animals. All the experiments were approved by the Committee on the Ethics of Laboratory Experiments of the Charles University in Prague and were performed under permission no. MSMT-31114/2013-13 of the Ministry of the Environment of the Czech Republic. Investigators are certificated for experimentation with animals by the Ministry of Agriculture of the Czech Republic.

### Parasites and vectors

*Leishmania enriettii* LV90 (MCAV/BR/45/LV90) and *Leishmania* sp. AM-2004 (MMAC/AU/2004/AM-2004; Roo1; LV756), and three human infecting *Leishmania* strains, *L*. *major* FVI (MHOM/IL/81/Friedlin/FVI), *L*. *infantum* CUK3 (TOB/TR/2005/CUK3) and *L*. *donovani* GR374 (MHOM/ET/2010/GR374), were used. Parasites were maintained at 23°C in M199 medium supplemented with 10% fetal calf serum (Gibco), 1% BME vitamins (Sigma), 2% sterile urine and 250 μg/ml amikin (Amikin, Bristol-Myers Squibb), and were in culture for about 10 subpassages from an animal host before use. Before experimental feeding, parasites were washed by centrifugation and resuspended in saline solution.

*Lutzomyia longipalpis* (Jacobina colony) was maintained at Charles University in Prague under standard conditions [29]. Females from the colonies of *Culicoides nubeculosus* and *C*. *sonorensis* (both belonging to subgenus *Monoculicoides*) were sent to Charles University from the Pirbright Institute, UK and kept at 20°C before exposure to feeding. All insects were initially given free access to 50% sucrose supplemented with penicillin (5000 U/ml), which was replaced with sugar solution alone 3 days before experimental feeding.

### Membrane feeding on infected blood

All infection experiments were performed at Charles University in Prague. In each experiment, approximately 150 *Lu*. *longipalpis* or *Culicoides* females (5–7 days old) were fed through a chick-skin membrane on heat-inactivated rabbit blood containing 10^7^ promastigotes/ml from one of the strains described above. Engorged females were separated, maintained at 26°C or 20°C, according to experimental design, and dissected at days 1–2, 3, 5–6 and 9–10 post-blood meal (PBM). The localization and intensity of *Leishmania* infection in guts were evaluated *in situ* under a light microscope, by scoring the proportions of flies with low (<100 parasites/gut), moderate (100–1000 parasites/gut) or heavy (>1000 parasites/gut) infections [30]. All experiments were repeated at least twice.

### Morphological analysis

Smears from midguts of *C*. *sonorensis* (7 and 10 days PBM) and *Lu*. *longipalpis* (10 days PBM) infected with *L*. *enriettii* were fixed with methanol, stained with Giemsa, examined under the light microscope with an oil-immersion objective and measured using ImageJ program. Body length, flagellar length and body width of parasites were measured for determination of morphological forms according to the criteria of Walters et al. [31] and Cihakova and Volf [32]. The following morphological forms were distinguished: (i) short nectomonads: body length <14 μm and flagellar length < 2 times body length; (ii) long nectomonads: body length ≥ 14 μm and (iii) metacyclic promastigotes: body length <14 μm and flagellar length ≥ 2 times body length, as summarized in Sadlova et al. [33].

### Infection and xenodiagnoses of guinea pigs and hamsters

Two guinea pigs (*Cavia porcellus*) and two golden hamsters (*Mesocricetus auratus*), anaesthetized with ketamin/xylazin (150 mg/kg and 15 mg/kg, respectively), were injected with 10^7^ late-log stage promastigotes intradermally into the ear pinnae and nose. The course of infection was recorded weekly.

Xenodiagnoses were performed on animals 3, 4, 7, 9, 12 weeks post-infection (PI) using *Lu*. *longipalpis* (5–6 days old) and *C*. *sonorensis* (5 days old). Female *Lu*. *longipalpis* or *C*. *sonorensis* were placed into plastic vials covered by fine nylon mesh and allowed to feed on the inoculated site of anaesthetized animals. Successfully blood-fed individuals were then maintained for two days at 20°C and then stored in Elution Buffer at -20°C for subsequent quantitative PCR (Q-PCR). After the last xenodiagnosis, the hosts (golden hamsters and guinea pigs) were euthanized, dissected and tissues from ears, draining lymph nodes, noses, spleens, livers and blood were stored at -20°C for subsequent Q-PCR.

### Quantitative PCR

Extractions of DNA from vectors and animal tissues were performed using a High Pure PCR Template Preparation Kit (Roche) according to the manufacturer´s instructions. The total DNA was used as a template for Q-PCR amplification with the primers described by Mary et al. [34] in Bio-Rad iCycler and iQ Real-Time PCR Systems using the SYBR Green detection method (iQ SYBR Green Supermix, Bio-Rad).

## Results

### Development of *L*. *enriettii* and *Leishmania* sp. AM-2004 in *Lu*. *longipalpis*, *C*. *nubeculosus* and *C*. *sonorensis*

**Fig 1:**
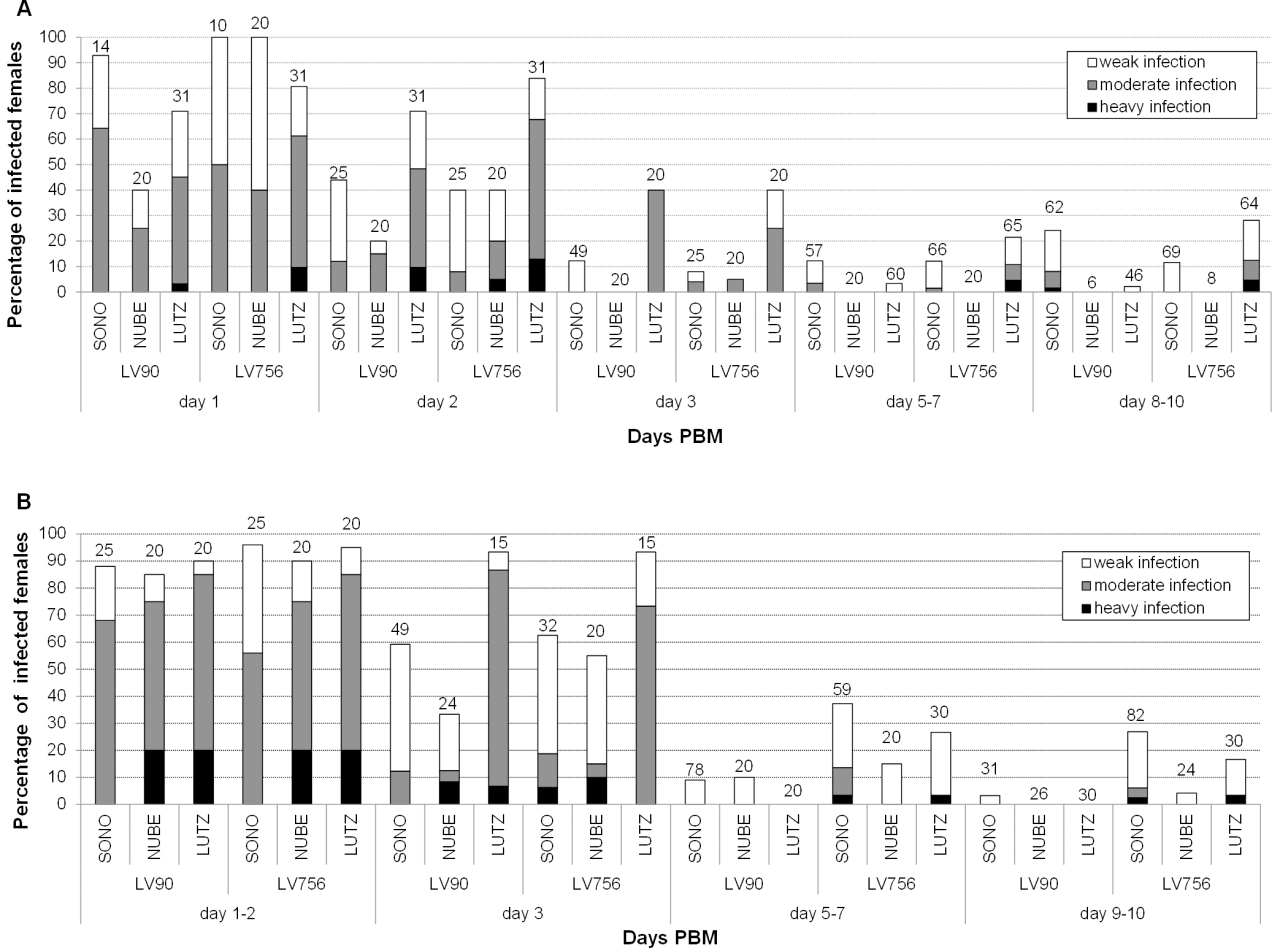
Development of *Leishmania* from the *L*. *enriettii* complex in vectors. Experimental infection of the sand fly *Lutzomyia longipalpis* (LUTZ) and two biting midges *Culicoides nubeculosus* (NUBE) and *C*. *sonorensis* (SONO) with *Leishmania enriettii* (LV90) and *Leishmania* sp. AM-2004 (LV756). Insects were maintained either at 26°C (A) or 20°C (B). Intensities of infection were estimated as light (˂100 promastigotes/gut), moderate (100–1,000 promastigotes/gut) or heavy (˃1,000 promastigotes/gut). Numbers above each bar indicate the number of dissected females.

Infection of *Lu*. *longipalpis* was attempted with two species of *Leishmania*, *L*. *enriettii* (LV90 strain from Brazil) and *Leishmania* sp. AM-2004 (LV756 strain from Australia), by membrane feeding in flies maintained at two different temperatures (26°C and 20°C). At 26°C (Fig 1A), a high infection rate (70–80%) was observed for both parasite species on days 1–2 PBM, all parasites being located in the abdominal midgut (AMG). Then, due to defecation of the blood meal remnants, the infection rate was reduced to 40% on day 3 PBM. In late stage infections (days 5–10 PBM), *L*. *enriettii* was observed only at low numbers, all being located in the AMG with no colonisation of the stomodeal valve (SV). *Leishmania* sp. AM-2004 generated somewhat better infections, producing moderate or heavy infections in 12% of infected females and colonization of the SV in 15–20% of them. At 20°C parasite development was similar (Fig 1B), but no *L*. *enriettii* and very few *Leishmania* sp. AM-2004 infections developed to a late-stage in *Lu*. *longipalpis*.

In *C*. *nubeculosus*, *L*. *enriettii* and *Leishmania* sp. AM-2004 parasites were present only in the AMG before and immediately after defecation. On days 6 and 10 PBM, all 54 examined females maintained at 26°C were negative (Fig 1A), while in those maintained at 20°C very few parasites were occasionally found in the abdominal midgut (Fig 1B). Neither *Leishmania* species were able to establish late stage infections in *C*. *nubeculosus* or any colonization of the SV.

In *C*. *sonorensis*, *L*. *enriettii* and *Leishmania* sp. AM-2004 both developed early stage infections at high rates (in approximately 90% of midges), producing mostly moderate infections (Fig 1A and 1B). Immediately after defecation (2 days PBM at 26°C and 3 days PBM at 20°C), again the parasite numbers were reduced, but moderate or heavy infections were still observed in some females. However, a striking contrast to *C*. *nubeculosus* was observed in parasite development on days 5–7 PBM and onwards. The infection rates observed were comparable to *Lu*. *longipalpis*, however, in *C*. *sonorensis*, *Leishmania* promastigotes migrated to the thoracic midgut (TMG), forming typical rosettes, and then colonized the SV in 20–25% and 20–38% of midges, for *L*. *enriettii* and *Leishmania* sp. AM2004, respectively, which are significantly higher percentages than in *Lu*. *longipalpis* for both parasite species. Parasite development was similar and the rate of SV colonization was comparable at both temperatures tested (Fig 1A and 1B).

**Fig 2:**
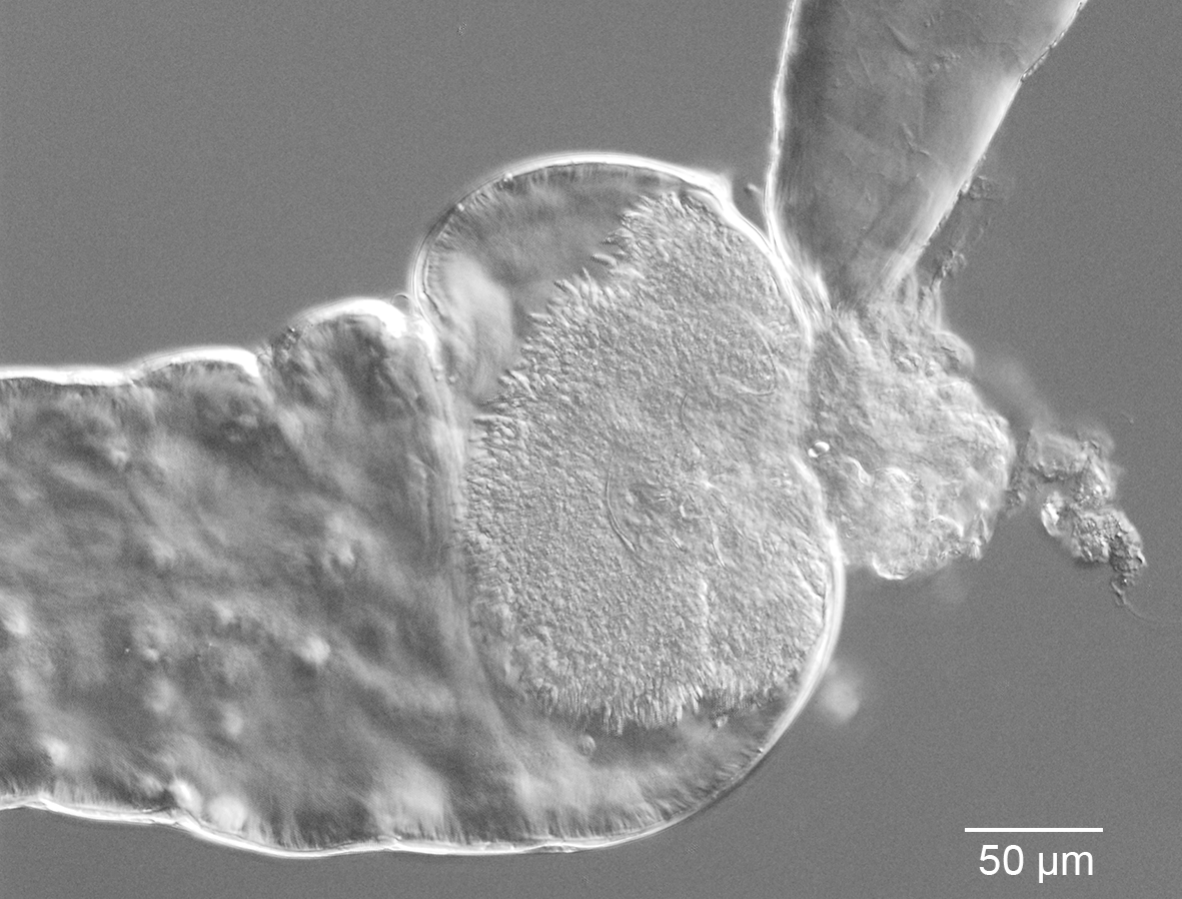
Midgut dissected from *C*. *sonorensis* females with infection of *L*. *enriettii* colonizing the stomodeal valve. Phase contrast light microscopy showing a mass of promastigotes attached to the stomodeal valve. Bar represents 50 μm.

**Fig 3:**
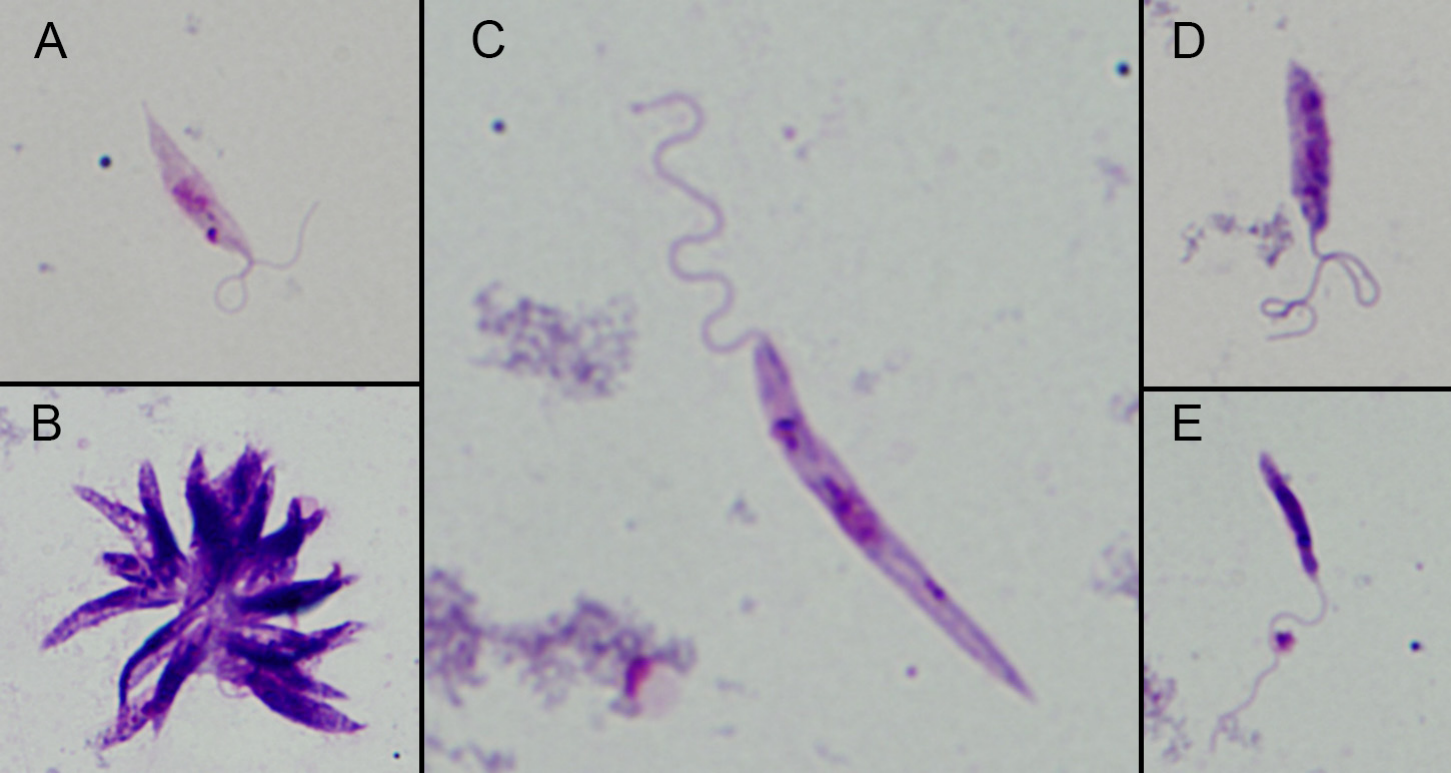
Morphological forms determined in midgut smears. *Leishmania* parasites distinguished in the midgut of *C*. *sonorensis* infected with *L*. *enriettii* 10 days PBM. (A) Short nectomonads, (B) short nectomonads forming in rossetes, (C) long nectomonads and (D and E) metacyclic forms.

Light microscopy was used to examine *L*. *enriettii* parasites in the region of the SV (Fig 2). Large masses of parasites could be seen attached to the cuticular surface of the SV, potentially partially obstructing the opening of the SV. Morphological analysis was performed on *L*. *enriettii* parasites recovered from *Lu*. *longipalpis* and *C*. *sonorensis* at 10 days PBM. The majority of parasites were short nectomonads, 80% in *Lu*. *longipalpis* and 72% in *C*. *sonorensis*, and many of these were in rosettes. There were also long nectomonads present, 13% and 23%, respectively, and metacyclic promastigotes at 7% and 5%, respectively (Fig 3).

### Development of *L*. *major*, *L*. *infantum* and *L*. *donovani* in *Culicoides sonorensis*

**Fig 4:**
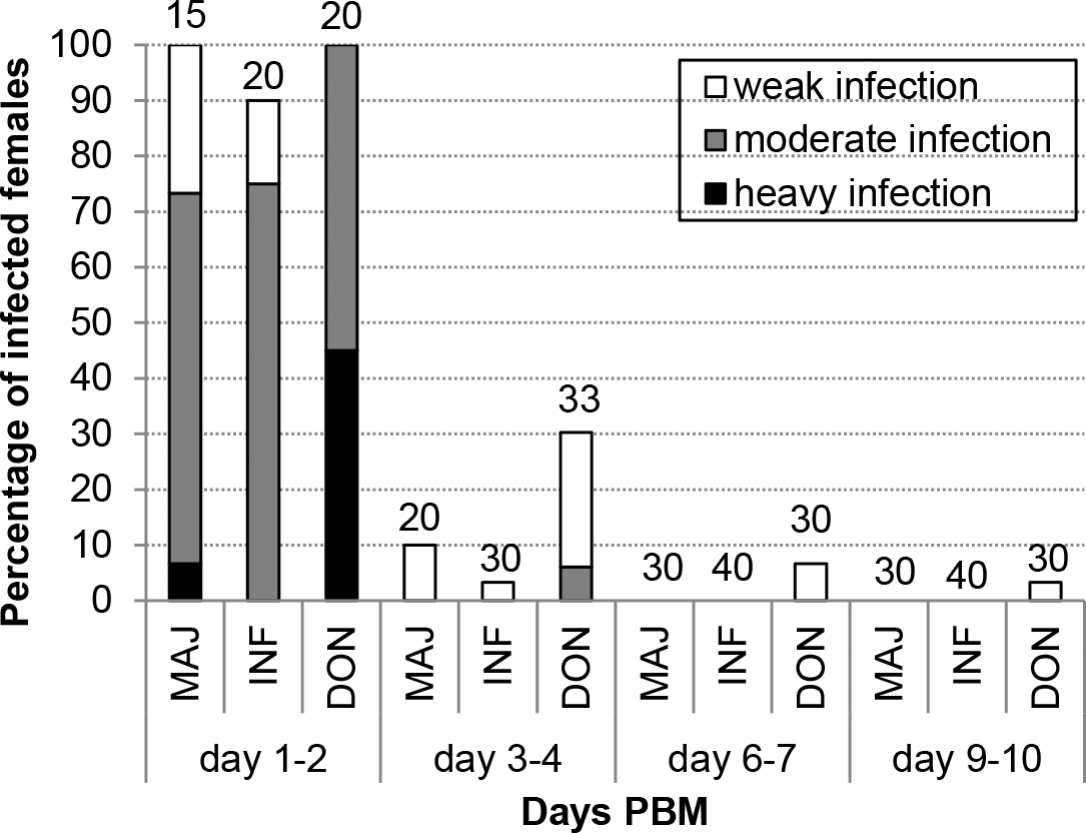
Development of human-infecting *Leishmania* species in *C*. *sonorensis*. Experimental infection of *C*. *sonorensis with L*. *major* FVI (MAJ), *L*. *infantum* CUK3 (INF) and *L*. *donovani* GR374 (DON) (at 20°C). Intensities of infection were estimated as in Fig 1.

To evaluate the significance of the above results with *L*. *enriettii* complex parasites in *C*. *sonorensis*, by membrane feeding we tested the susceptibility of this biting midge to three *Leishmania* species from the subgenus *L*. (*Leishmania*) capable of infecting humans, all of which have proven sand fly vectors. None of these *Leishmania* species were able to develop successfully into late stage infections in *C*. *sonorensis* (Fig 4). Before defecation of blood meal remnants, high numbers of procyclic promastigotes were found in the abdominal midgut in more than 90% of females on days 1–2 PBM. However, after defecation (day 3 PBM and onward) the majority of *C*. *sonorensis* females were negative and the rest had only very few parasites in the AMG. On days 6–10, no *L*. *major* or *L*. *infantum* were present in the midges, although three out of 60 females infected by *L*. *donovani* displayed long nectomonads in the AMG, but without any parasites in the TMG or SV (Fig 4). No metacyclic promastigotes were observed in *C*. *sonorensis* infected with *L*. *major*, *L*. *infantum* or *L*. *donovani*.

### Course of infection in guinea pigs and xenodiagnosis

**Fig 5:**
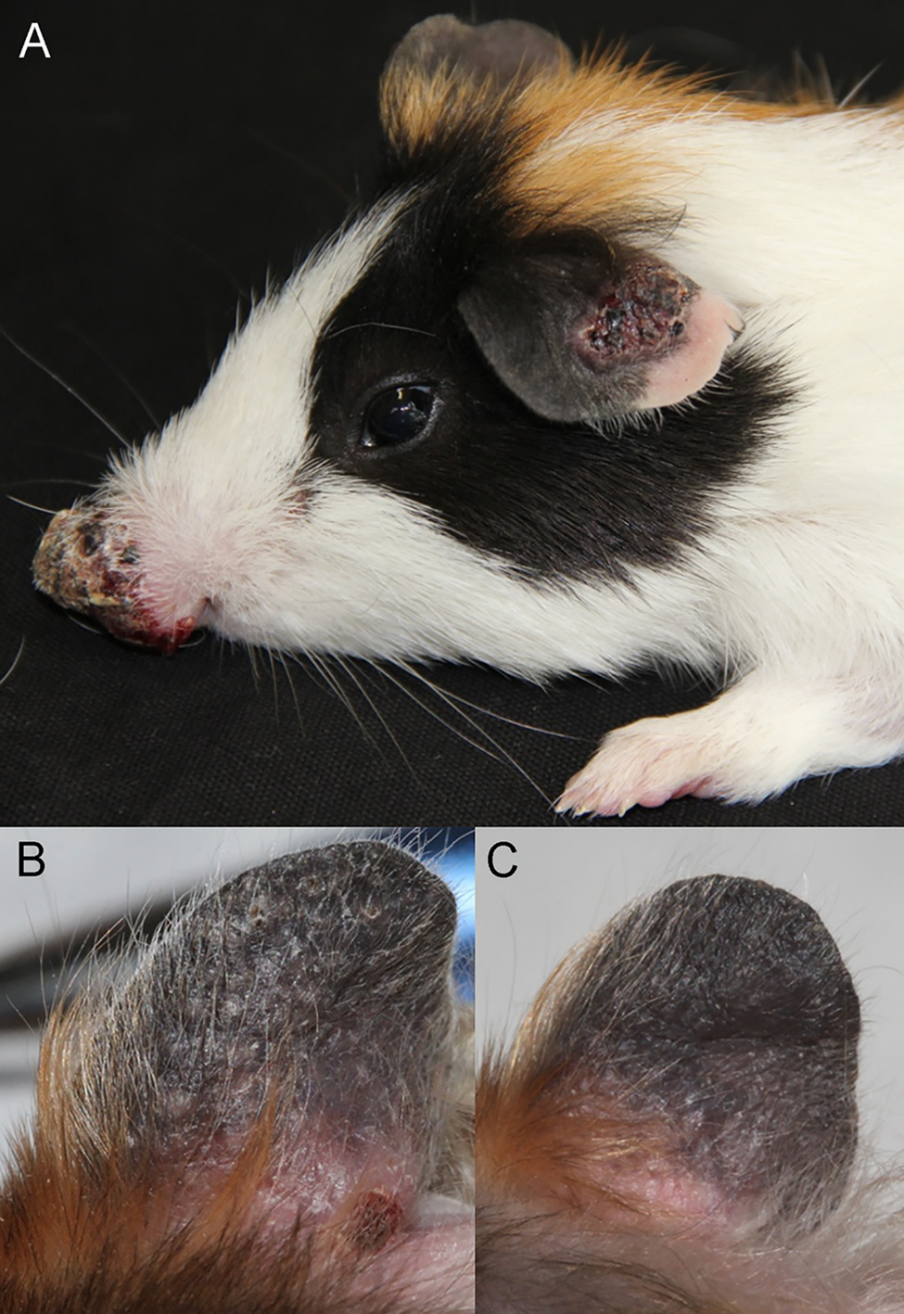
Skin symptoms in rodent hosts infected by *L*. *enriettii*. Manifestation of infection on the ear and on the nose of the guinea pig 12 weeks post infection (A). Same ear of the golden hamster 6 weeks post-infection (B) and 12 weeks post-infection (C).

The first clinical signs of infection with *L*. *enriettii* in guinea pigs were redness and swelling on the inoculated ear 3–4 weeks PI. In the following two weeks (5–6 weeks PI), the swelling developed into small cutaneous lesions (~4×3 mm), which later grew rapidly to become large and ulcerated (~14×10 mm) by weeks 9–12 PI. In addition, a secondary dermal lesion appeared in one guinea pig on the skin between the eyes and nose (4.1×4.7 mm). In the animal inoculated via a nasal route, the first clinical manifestation of infection was observed in week 5 PI (two weeks later than on the the ear), but then the signs increased in severity more rapidly and later resembled a necrotic tumour-like ulcer by the end of experiment (12 weeks PI) (Fig 5).

In total, 195 *Lu*. *longipalpis* and 125 *C*. *sonorensis* female adults were fed on the ears, and 93 *Lu*. *longipalpis* and 69 *C*. *sonorensis* fed on the noses of two guinea pigs infected with *L*. *enriettii*. Preliminary experiments confirmed that *Leishmania* in such insect guts transformed from amastigote to short and long nectomonads, metacyclic promastigote forms and that in *C*. *sonorensis* these proliferated vigorously and colonized the stomodeal valve. The development pattern was similar as observed during experimental feeding. Therefore, Q-PCR was an appropriate method for quantification of xenodiagnosis.

**Fig 6:**
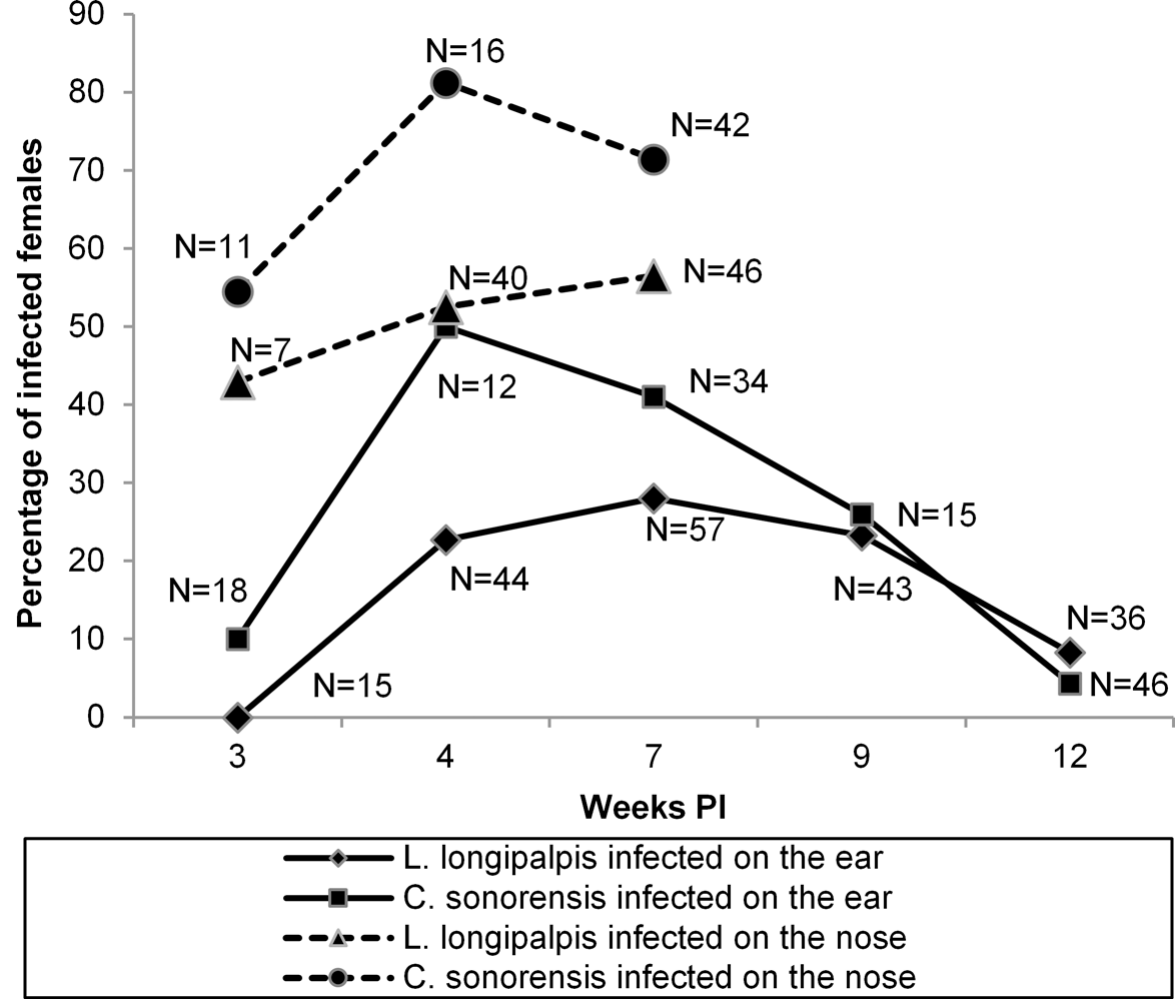
Infectiousness of guinea pigs for *Lu*. *longipalpis* and *C*. *sonorensis* feeding on *L*. *enriettii* inoculated ears or noses. Xenodiagnoses were performed at three intervals (3, 4, 7 weeks post-infection) on ears of guinea pigs, and five times (3, 4, 7, 9 and 12 weeks post-infection) on noses of guinea pigs. The fed females were tested for presence of *Leishmania* parasites 2 days PBM using Q-PCR.

Both infected ears and noses appeared to be a good source of parasites for the insects. In *C*. *sonorensis* the infection rates were about 50% and 80% for ears and nose, while in *Lu*. *longipalpis* the infection rates were a little lower at about 30% and 50%, respectively. In both vectors, the highest infection rates were observed using animals between 4–7 weeks PI, afterwards the infectivity of guinea pigs for both vectors decreased. The xenodiagnosis results in guinea pigs are summarized in Fig 6.

After the last xenodiagnosis experiment (12 weeks PI), the guinea pigs were euthanized and Q-PCR showed high numbers of parasites present in inoculated ears and noses (ranging from 8.8×10^6–^3.8×10^7^ parasites in each organ). In guinea pigs *L*. *enriettii* also visceralized to the spleen, and parasites were also detected in draining lymph nodes and co-lateral ears (20–500 parasites in each organ).

### Course of infection in hamsters and xenodiagnosis

In golden hamsters the first signs of disease (redness and swelling) were also observed 3 weeks PI on the inoculated ears. Then, however, the course of infections strikingly differed from those in guinea pigs. In hamsters, multiple small nodules appeared on the inoculated ears in weeks 4–7 PI (Fig 5B), but all these nodules self-healed after 7–9 weeks PI (Fig 5C). Similarly, on the nose the infection was manifested only by redness, small sores and oedema, which was reabsorbed by 7 weeks PI. At the end of the experiment neither hamster presented clinical signs of infection.

In total, 159 *Lu*. *longipalpis* and 112 *C*. *sonorensis* were fed on ears and noses of two hamsters infected with *L*. *enriettii*. Generally, the vectors were less willing to feed on hamsters than on guinea pigs and their infectivity rate was much lower. *Leishmania* were detected only in two groups of *Lu*. *longipalpis* females fed on hamster ears 4 and 9 weeks PI (infection rates were about 30% and 10%, respectively). No positivity was found in 80 *C*. *sonorensis* females fed on the hamster´s ears. All 85 *Lu*. *longipalpis* and 31 *C*. *sonorensis* fed on inoculated noses were negative. In hamsters euthanized 12 weeks PI the Q-PCR results demonstrated very low parasite numbers in inoculated ears (<50 parasites). Other organs tested (nose, co-lateral ear, draining lymph node, blood, spleen and liver) were negative in both hamsters.

## Discussion

To date phlebotomine sand flies are the only proven vectors of *Leishmania* species, however, based on the discovery of *Leishmania* sp. AM-2004 in Australian biting midges [5], we assessed the possibility that *L*. *enriettii* may also have a midge vector. Based on the examination of vector competence presented here, we conclude that it is more likely that *L*. *enriettii* is transmitted by biting midges than by sand flies. However, several important aspects of vector incrimination need to be tested in future work such as their ecological associations with reservoir hosts and transmission dynamics, which may either provide further support for midge-transmision or lead to rejection of this hypothesis. There are many neotropical species of midges and sand flies, and resolution of this will require careful fieldwork and laboratory testing of any new proposed midge or sand fly vector. In the meantime we recommend that vector studies on members of the *L*. *enriettii* complex consider both midges and sand flies as potential vectors.

Our conclusion that *L*. *enriettii* is most likely to be midge-transmitted is based on several lines of evidence presented here: *L*. *enriettii* developed in the three insects in a similar way to *Leishmania* sp. AM-2004 and in marked contrast to *L*. *major*, *L*. *infantum* and *L*. *donovani*; the best insect host for *L*. *enriettii* was *C*. *sonorensis*, showing a similar pattern of development to that seen in *Lu*. *longipalpis* but with a higher percentage of stomodeal valve infections, and surviving beyond the blood meal to a "late stage" infection; *L*. *major*, *L*. *infantum* and *L*. *donovani* did not survive after the bloodmeal in *C*. *sonorensis* but previous work has shown these to develop mature transmissible infections in *Lu*. *longipalpis*; and both *C*. *sonorensis* and *Lu*. *longipalpis* were infected after feeding on infected guinea pigs, but to a greater extent in *C*. *sonorensis*. These data are consistent with midge-transmission of *L*. *enriettii*, but do not prove it, and each is discussed in more detail below.

Colonization of biting midges is regarded as extremely challenging, as only a very small number of species possess life cycles traits suitable for laboratory maintanance and the vast majority will not take blood meals under laboratory conditions [25]. The Nearctic species *C*. *sonorensis* was demonstrated to be susceptible to infection and our experiments showed that *L*. *enriettii* developed late stage infections in 10–30% of *C*. *sonorensis* females. We define these as "late stage" infections, meaning that they have progressed beyond the early blood meal phase and become established in the midges. The development seen is remarkable and similar to that seen with *Leishmania* sp. AM-2004, but in marked contrast to *L*. *major*, *L*. *infantum* or *L*. *donovani*. Moreover, 20% of infected midges with such late stage *L*. *enriettii* infections exhibited heavy colonization of the SV. Short and long nectomonads were observed during the late-stage infection in *C*. *sonorensis* gut. The short nectomonads are responsible for forward migration and colonization of the stomodeal valve including production of promastigote secretory gel (PSG), which together with sand fly saliva are critical components for disease outcome and progress [35,36]. One area of interest for future investigation would be to see if midge saliva had disease exacerbating properties similar to those of sand fly saliva [37]. Localization of parasite masses on the SV and presence of metacyclic promastigotes is associated with *Leishmania* transmission in sand flies [38,39] and has been observed in *Forcipomyia* midges naturally infected with *Leishmania* sp. AM-2004 [5]. It should be noted that these experiments were performed by membrane feeding, where high doses of parasites can be ingested.

*Lu*. *longipalpis*, a widespread vector of *L*. *infantum* in Latin America, was capable of supporting *L*. *enriettii* to a similar extent as *C*. *sonorensis*, although fewer SV infections were observed. However, all other *Leishmania* species tested in *Lu*. *longipalpis* to date, including *L*. *major*, *L*. *infantum* and *L*. *donovani* produce mature infections with high precentages of metacyclic promastigotes and PSG [28]. The percentages and intensity of late stage infections observed here for *L*. *enriettii* are far lower than normally found for infection of *Lu*. *longipalpis* with other *Leishmania* species. The Palearctic species *C*. *nubeculosus* was not susceptible to *Leishmania enriettii*, but neither was it susceptible to *Leishmania* sp. AM-2004. This lack of vector competence is consistent with our previous findings that *C*. *nubeculosus* does not support development of *L*. *infantum* and *L*. *major* [40]. In fact this is the predicted outcome given that there are over 1400 known species of *Culicoides* known worldwide [25], so the chances of finding one that supports post-blood meal development of any *Leishmania* parasite must be quite low, and just further emphasises the potential significance of the results obtained with *C*. *sonorensis*.

*Leishmania enriettii* is known as a pathogen of guinea pigs causing tumour-like skin lesions. While some authors [41,42] reported metastatic spread of parasites to distant parts of the guinea pig body (eyelids, lips, feet and genitalia), others found parasites limited to the inoculation site [10,37]. The evolution of skin lesions caused by *L*. *enriettii* can be extremely fast within two weeks PI [42] and can be enhanced by addition of sand fly salivary gland extract to the inoculum [37]. In our study, disease manifestation differed between individuals and inoculation sites. Lesions developed 5 weeks PI on the ear (ulcerated between 7–9 weeks PI) and 7 weeks PI on the nose. However, parasites inoculated into the noses grew very quickly, producing large, ulcerated, tumour-like lesions. We did not observe a self-healing process, as previously reported [37,42,43]. According to recent studies, parasites of the *L*. *enriettii* complex do not only cause cutaneous forms of leishmaniasis, but can also produce visceral leishmaniasis [7,13,19]. These findings correlate with our results from Q-PCR, which detected *L*. *enriettii* parasites in the draining lymph nodes and spleen of infected guinea pigs.

In hamsters, *L*. *enriettii* is known to be less pathogenic than in guinea pigs and some studies suggested spontaneous self-healing [44]. Here, we demonstrated that experimental infection of hamsters led only to mild symptoms. On the ear, non-ulcerated multiple nodules appeared at four weeks PI, but had self-healed by eight weeks PI. No clear signs of disease were recorded on the nose during the entire experimental period. This is in accordance to results from xenodiagnosis showing that experimentally infected hamsters were less infectious, with a low proportion of infections found in female *Lu*. *longipalpis* fed on ears 4 and 9 weeks PI, but no infections were seen in *C*. *sonorensis*.

Xenodiagnosis is currently the gold standard method used to determine infectivity of naturally or experimentally infected hosts for insect vectors. It has been repeatedly used to prove infectivity of potential reservoirs to natural vectors of *L*. *infantum* [45–47] and *L*. *tropica* [48]. In the current study the infection rate recorded was up to 50% in *Lu*. *longipalpis* and up to 80% in *C*. *sonorensis*. This is a much higher infection rate than achieved using any rodent infected with *L*. *major*, *L*. *tropica* or *L*. *donovani* [48,49]. Similar high rates (around 60–80%), were obtained only using *P*. *perniciosus* and *Lu*. *longipalpis* fed on *L*. *infantum*-infected dogs [50,51]. It also demostrated that guinea pigs were most infective for *Lu*. *longipalpis* and *C*. *sonorensis* one month post-infection, despite more serious clinical manifestation of the disease being found later during the experiment. These results agree with previous findings using mouse models where no direct link was observed between host symptoms and infectivity to vectors [48,49].

In summary, we have demonstrated experimentally, for the first time, that two species of the *L*. *enriettii* complex, *L*. *enriettii* and *Leishmania* sp. AM-2004, can develop late-stage infections in the biting midge *C*. *sonorensis*. This species provides a readily manipulable experimental subject for study of the *L*. *enriettii* complex under laboratory conditions; it was found to be similarly susceptible to these parasites as a permissive sand fly species *Lutzomyia longipalpis*. Both promastigote and amastigote infection of *C*. *sonorensis* (performed by membrane feeding and xenodiagnoses, respectively) resulted in masses of parasites in thoracic midgut and colonization of the stomodeal valve, which was found twice as frequently in *C*. *sonorensis* as in *Lu*. *longipalpis*. These data support those of Dougall et al. [5] who reported mature infections of *Leishmania* sp. AM-2004 in field-collected biting midges of the genus *Forcipomyia*. Our results support the hypothesis that biting midges might be natural vectors of the *L*. *enriettii* complex, but more detailed studies especially focused on transmission potential and field collections need to be done. However, these results should be taken in consideration while searching for vectors of *L*. *martiniquensis*, "*L*. *siamensis*" and the recently reported species from Ghana, whose sand fly vectors are unknown.
